# Does Consideration and Assessment of Effects on Health Equity Affect the Conclusions of Systematic Reviews? A Methodology Study

**DOI:** 10.1371/journal.pone.0031360

**Published:** 2012-03-13

**Authors:** Vivian Welch, Mark Petticrew, Erin Ueffing, Maria Benkhalti Jandu, Kevin Brand, Bharbhoor Dhaliwal, Elizabeth Kristjansson, Janet Smylie, George Anthony Wells, Peter Tugwell

**Affiliations:** 1 Clinical Epidemiology Unit, Ottawa Hospital Research Institute, Ottawa Hospital, Ottawa, Ontario, Canada; 2 Centre for Global Health, Institute of Population Health, University of Ottawa, Ottawa, Ontario, Canada; 3 London School of Hygiene and Tropical Medicine, London, United Kingdom; 4 Campbell and Cochrane Equity Methods Group, Centre for Global Health, Institute of Population Health, University of Ottawa, Ottawa, Ontario, Canada; 5 Telfer School of Management, University of Ottawa, Ottawa, Ontario, Canada; 6 School of Psychology, University of Ottawa, Ottawa, Ontario, Canada; 7 Centre for Research on Inner City Health, Keenan Research Centre of the Li Ka Shing Knowledge Institute, St. Michael's Hospital, Toronto, Ontario, Canada; 8 Dalla Lana School of Public Health, University of Toronto, Toronto, Ontario, Canada; 9 Department of Epidemiology and Community Medicine, University of Ottawa, Ottawa, Ontario, Canada; 10 University of Ottawa Heart Institute, Ottawa, Ontario, Canada; 11 Department of Medicine, University of Ottawa, Ottawa, Ontario, Canada; University Paris Descartes, France

## Abstract

**Introduction:**

Tackling health inequities both within and between countries remains high on the agenda of international organizations including the World Health Organization and local, regional and national governments. Systematic reviews can be a useful tool to assess effects on equity in health status because they include studies conducted in a variety of settings and populations. This study aims to describe the extent to which the impacts of health interventions on equity in health status are considered in systematic reviews, describe methods used, and assess the implications of their equity related findings for policy, practice and research.

**Methods:**

We conducted a methodology study of equity assessment in systematic reviews. Two independent reviewers extracted information on the reporting and analysis of impacts of health interventions on equity in health status in a group of 300 systematic reviews collected from all systematic reviews indexed in one month of MEDLINE, using a pre-tested data collection form. Any differences in data extraction were resolved by discussion.

**Results:**

Of the 300 systematic reviews, 224 assessed the effectiveness of interventions on health outcomes. Of these 224 reviews, 29 systematic reviews assessed effects on equity in health status using subgroup analysis or targeted analyses of vulnerable populations. Of these, seven conducted subgroup analyses related to health equity which were reported in insufficient detail to judge their credibility. Of these 29 reviews, 18 described implications for policy and practice based on assessment of effects on health equity.

**Conclusion:**

The quality and completeness of reporting should be enhanced as a priority, because without this policymakers and practitioners will continue lack the evidence base they need to inform decision-making about health inequity. Furthermore, there is a need to develop methods to systematically consider impacts on equity in health status that is currently lacking in systematic reviews.

## Introduction

Health inequities have been defined as unfair and avoidable inequalities in health across socioeconomic, demographic and geographic factors [1]. Specific population groups have been identified as “vulnerable” to health inequities. This vulnerability in turn is linked to social systems that differentially distribute resources across sociodemographic strata such as gender, socioeconomic position, and ethnicity. Health inequity exists both within and between countries. For example, under five childhood mortality is less than 6 per 1000 births in industrialized countries compared to 160 per 1000 in sub-Saharan Africa [2]. Within low and middle income countries (LMIC), under-five mortality is a median of two times higher in the poorest people compared to the highest wealth quintile. Within high income countries, health inequalities also exist. For example, Aboriginal people in Canada have 5 years lower life expectancy than non-Aboriginals [3].

We have shown with the Equity Effectiveness Loop framework that the effectiveness of clinical and public health interventions may be reduced by up to two thirds in vulnerable populations. This is due to a “staircase” effect due to lower coverage, worse screening, poor provider compliance and lower consumer adherence [4]. Because of this staircase effect, interventions may have the unintended adverse effect of increasing inequity in health status.

Decision-makers are increasingly under public pressure to consider the effects of programs and policies on health equity [5], [6]. There is increasing acceptance that systematic reviews can inform policy and practice since they reduce the chance of being misled, increase confidence in results, are an efficient use of time and are more easy to critically appraise and apply [7], [8]. Systematic reviews also represent an opportunity to identify what works to reduce health inequity [5]. However, decision-makers cite lack of evidence on impacts of health interventions on equity as a major barrier to using systematic reviews for evidence-informed decision-making [9], [10].

We have a moral obligation to ensure that clinical and public health interventions help the disadvantaged, but all too often interventions justified in the name of the poor benefit the most advantaged people, not the poorest [11]. NICE in the UK has taken a leadership role in insisting that equity evidence is needed to inform decisions about the likely impacts of clinical and public health interventions in different population groups. Specifically, the 2009 NICE Public Health methods guidance asks “How does effectiveness and cost-effectiveness vary according to the age, gender, class and ethnicity of the target audience? Is there any differential impact on inequalities in health within and between different population groups?” [2]. Furthermore, the World Health Organization Commission on Social Determinants of Health and Campbell and Cochrane Equity methods group have proposed that more attention on health equity in systematic reviews could increase relevance for policy and clinical practice [12].

Despite this need to assess the evidence on impacts of health interventions on equity in health status, systematic reviews rarely assess whether interventions have an impact on health equity[13]. Furthermore, our Cochrane review of methods found that none of the included studies had assessed the credibility of subgroup analyses nor the importance of equity assessment for the implications for practice and policy [14]. Lack of credibility and failure to discuss implications are a substantial barrier in using systematic reviews for policy and practice decisions.

This methodology study aimed to describe the extent to which impacts of health interventions on equity in health status are considered in systematic reviews, describe methods used, assess their credibility and assess the implications of their equity-related findings for policy, practice and research.

The specific objectives were to: 1) Evaluate definitions of health equity in systematic reviews; 2) Assess methods used by systematic reviews to assess impacts of health interventions on equity in health status; 3) Assess subgroup analyses according to seven credibility criteria; and 4) Assess implications of equity findings on conclusions.

## Methods

Ethical approval was not required for this study.

### Definition of health equity

As above, health equity is defined as unfair and avoidable health differences [1]. The acronym PROGRESS-Plus defines sociodemographic factors across which differences in effectiveness of interventions could be considered inequitable [15], [16]: Place of residence; Race/ethnicity/culture; Occupation; Gender/sex; Religion; Education; Socioeconomic status; Social capital. The “Plus” was proposed to promote the inclusion of additional factors across which disadvantage may exist due to discrimination and other reasons such as age, sexual orientation and disability [17].

Differences in health status outcomes across PROGRESS-Plus were considered differences in equity in health status if they were classified as inequitable or unfair by the authors of the systematic review, or they met the Whitehead criteria for inequity in health [1].

### Study design

This study is designed as a methodology study, defined by the Cochrane Methodology Review group as: “*a study of the methods used in randomized trials, other healthcare evaluations or systematic reviews. Data for methodology studies can come from clinical studies, such as randomized trials, epidemiological studies, from participants in a new study, or from systematic reviews of clinical studies”* [18].

This study assessed the methods used in systematic reviews of effectiveness to consider effects on health equity.

### Data source

We selected all systematic reviews indexed on MEDLINE in one month (November 2004) because the characteristics of these systematic reviews were already well described by Moher et al [19], and we wanted a group of greater than 100 systematic reviews with a diversity of health conditions and interventions since we expected less than 10% of systematic reviews would assess effects on health equity. We previously found that there was no increase in equity analyses over time in systematic reviews between 2004 and 2008 (unpublished data).

This group of systematic reviews was assembled using Montori's empirical search terms for high sensitivity (>98%) in retrieval of systematic reviews [20]. The search was run on February 18, 2005, with the terms: (1) 200411$.ed; (2) limit 1 to English (3) 2 and (cochrane database of systematic reviews.jn. or search.tw. or metaanalysis.pt. or medline.tw. or systematic review.tw. or ((metaanalysis.mp,pt. or review.pt. or search$.tw.) and methods.ab.)). Two reviewers screened the titles and abstracts, then a single reviewer screened the full-text to identify articles which met the following definition for a systematic review: “the authors stated objective was to summarize evidence from multiple studies and the article described explicit methods” [19]. A second reviewer independently screened a random sample of 10% of the full-text reviews. The search was limited to English-language due to resource implications of including non-English articles [19]. There is no evidence from two previous studies that non-English language papers are likely to assess impacts on equity in health status differently or more frequently [13], [21]. The search retrieved 300 systematic reviews.

For the purpose of this methodology study on health equity, systematic reviews were included if the stated purpose was to assess the effects of an intervention on health outcomes. Adhering to this criterion resulted in the exclusion of 76 of the 300 systematic reviews. Reasons for exclusion fell into two main categories. Either the study did not assess the effectiveness of an intervention on health outcomes or the study was not concerned with health outcomes (e.g. focused on literacy instead of health).

### Data extraction for this study

Two reviewers (two of BD, EU, VW, MBJ) independently extracted data on reporting and analysis of differences in effectiveness across PROGRESS-Plus factors, using a pre-tested data extraction form. This data extraction form included 51 items on the characteristics of the population, intervention, comparison (setting), outcomes and study design (i.e. randomized controlled trials, observational studies or both), whether health inequalities or health inequities were described and how they were defined. Subgroup analysis across PROGRESS-Plus was assessed, using the definition in the Cochrane Handbook: “Subgroup analyses involve splitting all the participant data into subgroups, often so as to make comparisons between them. Subgroup analyses may be done for subsets of participants (such as males and females), or for subsets of studies (such as different geographical locations)” [22].

Extraction by two independent extractors was compared and differences were resolved by discussion. There were few discrepancies between data extractors.

#### Objective 1

We assessed whether health equity or health inequalities were defined and described in the systematic reviews. We looked for any description of how health equity was defined such as whether differences were avoidable or unfair, whether proxy measures were used (such as receipt of health insurance for the poor) and how the judgment of fairness or avoidability was made. We expected such definitions only for those reviews where the review intended to assess effects on health equity across one or more PROGRESS-Plus factor.

#### Objective 2

We assessed the methods used by systematic reviews to assess impacts of health interventions on equity in health status

#### Objective 3

Subgroup analyses conducted across PROGRESS-Plus factors were assessed according to the seven ‘credibility’ criteria for subgroup analysis, proposed by Oxman and Guyatt [23], that are in the Cochrane Handbook of Systematic Reviews [22]. These credibility criteria are intended to minimize the over-interpretation of spurious differences: 1. Clinically important difference? 2. Statistically significant difference? 3. A priori hypothesis? 4. Subgroup analysis one of small number of hypotheses tested? 5. Difference suggested by comparisons within studies? 6. Difference consistent across studies? 7. Indirect evidence that supports hypothesized difference?

#### Objective 4

We assessed whether differences in equity in health status across PROGRESS-PLUS factors were described in the discussion section and the implications for research and practice.

## Results

### Characteristics of included systematic reviews

Of the group of 300 systematic reviews, 224 were classified as assessing the effects of an intervention on health outcomes (web appendix S1). Of the 76 systematic reviews that were excluded, 16 assessed test characteristics of diagnostic methods, 21 conducted systematic reviews of research methods (e.g. quality assessment) and 39 assessed the association of patients' characteristics with outcomes.

Of the 224 included systematic reviews, 153 (68%) described the characteristics of the populations in the primary studies across one or more PROGRESS-Plus characteristics as follows: gender/sex (49%), age (47%), place of residence (22%), and LMIC setting (9%) were most frequently reported (Table 1), followed by race/ethnicity/culture (4%), socioeconomic status (3%), occupation (1%), education (1%) and social capital (1%).

**Table 1:** PROGRESS-Plus factors described in 224 systematic reviews.

	Population described by PROGRESS -Plus	Theory considers PROGRESS-Plus	Analysis of differences across PROGRESS-Plus (using qualitative, quantitative or targeted methods)	Applicability or implications consider PROGRESS-Plus
	68%(153/224)	8%(18/224)	12%(29/224)	21%(49/224)
Overall	153	18	29	49
Place	49	2	0	1
Race/ethnicity	10	4	2	9
Occupation	2	0	0	1
Gender/sex	109	3	9	11
Religion	0	0	1	0
Education	2	2	1	3
Socioeconomic status	6	3	7	7
Social capital	2	2	4	5
LMIC	21	3	3	15
Disability	3	3	3	13
Age	105	2	2	1

PROGRESS-Plus: Place of residence (including urban/rural); Race/ethnicity/culture, Occupation, Gender, Religion, Education, Socioeconomic status, Social capital; Plus includes age, disability and low and middle income country (LMIC).

**Note**: PROGRESS-Plus items add up to more than the overall because some systematic reviews assessed more than one PROGRESS-Plus factor.

### Objective 1: Definition of health inequalities

No reviews explicitly mentioned health equity. A small proportion (18/224) did describe in the background or discussion section that one or more PROGRESS-Plus factors were hypothesized to modify the impact of interventions. These 18 systematic reviews stated that health status differences across PROGRESS-Plus factors have been hypothesized elsewhere to affect the effectiveness of interventions, but none of these systematic reviews attached the term “health inequity” to these differences, nor hinted at this concept by describing these differences in health as unfair and avoidable.

### Objective 2: Methods used to assess differences in effects across PROGRESS-Plus equity determining variables

Of 224 systematic reviews, 29 assessed differences in effects across PROGRESS-Plus variables, using explicit methods of subgroup analysis (n = 15) or targeted analysis of vulnerable populations (n = 14) (Web appendix S2). None of the reviews used a gradient approach, defined as assessing differences in health across the whole range of a measure of disadvantage such as across income levels or grades of socioeconomic position, thus recognizing a systematic pattern of worsening health outcomes with greater disadvantage [24] Nine of these 29 systematic reviews described health inequalities which were hypothesized to influence the effectiveness of interventions.

Of these 29 systematic reviews, 24 found differences in effects of interventions on health or social outcomes across PROGRESS-Plus variables.

### Objective 3: Assess subgroup analyses according to seven credibility criteria for subgroup analyses

Subgroup analyses were classified into two types: 1) pooled, and 2) description of within-study differences. Seven systematic reviews compared pooled results from more than one study using statistical methods. Eight systematic reviews described differences in effects across one or more PROGRESS-Plus factor within individual studies, without combining data.

#### a) Pooled results (n = 7 systematic reviews)

Seven systematic reviews assessed impacts of health interventions on equity in health status using subgroup analysis. These subgroup analyses met a median of three out of the seven credibility criteria (min-max 2–5) (Table 2). These seven systematic reviews assessed differences in relative effects (n = 2) and absolute effects (n = 5). Only one of these seven systematic reviews found a statistically significant difference; between studies of educational interventions which reported the gender ratio and those that did not [25]. According to the authors of the systematic review, this result may be related to lack of reporting rather than true differences between boys and girls [25].

**Table 2:** Characteristics of subgroup analyses in systematic reviews which combined data from different studies across PROGRESS-Plus factors, according to Oxman and Guyatt credibility criteria for subgroup analyses.

	PROGRESS-Plus factor	Method to compare difference	Size of difference in effects	95% CI of difference in effects	Statistics used to compare groups	1. clini-cally important difference?	2. statisti-cally significant difference?	3. a priori hypothesis	4. one of small number of hypotheses?	5. differences suggested by within study compare-sons-	6. differ-ence consist-ent across studies?	7. indirect evidence to support hypothesis?
[29]	Gender	Relative	0.22 difference in relative risk	N.d.	N.d.	N	N	Y	Y	Y	N.d.	N
[30]	LMIC	Relative	0.37 difference in relative risk	N.d	N.d	Y	N.d.	Y	Y	N	N	Y
[31]	Ethnicity	Absolute	13–20% difference in sustained viral response	Not done	Not done	N.d.	N.d.	Y	Y	Y	Y	Y
[32]	SES	Absolute	23% difference in preventive fraction	Not done	Chi square	Y	N.d.	Y	Y	N	N	Y
[25]	Gender	Absolute	0.48 difference in effect size between studies which report gender ratio and those that do not	N.d.	N.d.	N	Y	Y	N	N.d.	N.d.	N.d.
[33]	SES	Absolute	Effect size larger for those with low baseline nutritional status	N.d	N.d	N	N	Y	Y	Y	N	N.d.
[34]	Gender	Absolute	No difference	Not done	N.d.	N	N	Y	N	N	N	Y

LMIC- low and middle income countries, SES: socioeconomic status, n.d.- not described.

#### b) Description of within-study differences without pooling (n = 8)

Eight systematic reviews described differences in effects across PROGRESS-Plus characteristics observed within individual studies (Web Appendix S2). These systematic reviews described the effects within individual studies across gender/sex (n = 7), socioeconomic status (n = 3), education (n = 1), occupation (n = 1), religion (n = 1) and age (n = 2).

### Objective 4: Influence of considering health equity on conclusions

Of those 29 reviews which evaluated effects on equity in health status, 18 (62%) described implications for policy, practice or research (Web Appendix S2).

## Discussion

This study of 224 systematic reviews is the largest study of equity assessment in a random sample of systematic reviews, and it is the first study to assess the reporting of subgroup analyses related to vulnerable populations using explicit criteria and to assess the influence of equity analyses on the implications and conclusions of reviews. This study found that subgroup analyses were not reported in sufficient detail to judge their credibility. Secondly, this study found that equity results influenced the implications for two thirds of reviews.

We found that 13% of systematic reviews assessed effects of interventions in vulnerable populations using either subgroup analysis or targeted approaches, and 68% of systematic reviews described the population according to one or more PROGRESS-Plus factor. We propose that description of populations for some characteristics of PROGRESS-Plus, such as age and sex, is necessary to adhere to reporting standards and does not imply an equity perspective. However, some characteristics such as socioeconomic status and low and middle income country setting may indeed suggest an equity perspective is taken, and this would increase the proportion of systematic reviews with an equity perspective to higher than 13%.

None of the systematic reviews assessed whether effects were different across a gradient of disadvantage. None of the systematic reviews used the term “health equity” or described differences in effects as unfair. This is not necessarily undesirable and may reflect uncertainty about the normative judgment of fairness in different settings. For those systematic reviews that conducted subgroup analysis, the methods for reporting them met only three out of seven criteria for assigning credibility to a subgroup analysis [23].

This study has a number of strengths. We performed double extraction with verification and comparison of results and a pre-tested extraction form developed using published tools and guidance. This random sample of systematic reviews was assembled with transparent search criteria. The reporting characteristics of these systematic reviews are given elsewhere [19]. We identified that there was no time trend in planned or conducted subgroup analyses across PROGRESS-Plus factors from 2004 to 2008, thus justifying the use of this sample from November 2004.

The weaknesses of this study are that we did not assess the availability of data on effects in vulnerable populations from the primary studies. Three other studies have found that approximately 10% of primary studies report data disaggregated by one or more of the PROGRESS-Plus factors [13], [21], [26]. We addressed this weakness by explicitly assessing whether systematic reviews described availability of data in primary studies. Another limitation is that this methodology study was limited to systematic reviews indexed in MEDLINE. Since we aimed to assess effects of health interventions, this was an appropriate sample for our study. However, systematic reviews in non-medical databases such as Campbell Collaboration reviews may be more likely to assess differences in effects across socioeconomic status because they may focus on more upstream interventions [27], [28]. This is unlikely a serious limitation, and very unlikely to affect the conclusions of this study. Another limitation is that we excluded non-English systematic reviews. In two previous methods study, non-English studies were not qualitatively or quantitatively different from English studies in how they considered health equity or reported results [13], [21]. Thus, it is unlikely that this exclusion would affect the conclusions of this study.

We did not assess whether subgroup analyses that were planned were not reported or not conducted due to insufficient data or non-statistically significant results or other factors. Thus, there may be more equity subgroup analyses conducted that are not statistically significant and would not have an influence on the conclusions. In a prior study, we found that approximately half of planned subgroup analyses were not reported in the results (unpublished data).

### Conclusions

Our findings suggest that there is inadequate consideration of effects on health equity in systematic reviews. We propose that the decision about whether to assess health equity needs to be informed by the theory underlying the intervention, and that this needs to be described in systematic reviews. Subgroup analyses are only one way that effects on health equity can be assessed and they are subject to spurious results and may be misleading. Applicability and targeted approaches are also useful for considering effects on health equity. The quality and completeness of reporting should be enhanced as a priority, because without this policymakers and practitioners will continue lack the evidence base they need to inform decision-making about inequity. However it is not only about quality of reporting, it is about the fundamental need to find a way to systematically consider impacts on equity in health status in a way that is currently missing from systematic review processes. The Campbell and Cochrane Equity Methods Group is developing data-driven guidance on these methods for systematic review authors and users.

## Supporting Information

Web Appendix S1 **224 included systematic reviews.**
(DOC)

Web Appendix S2 **Systematic reviews (n = 29) which assessed differences across PROGRESS-Plus by: 1) subgroup analysis; 2) targeted; or 3) description of individual studies.**
(DOCX)
