# Supplementary material for: Does Consideration and Assessment of Effects on Health Equity Affect the Conclusions of Systematic Reviews? A Methodology Study
Source: PLoS One. 2012 Mar 13;7(3):e31360. doi: 10.1371/journal.pone.0031360 (PMC3302723; doi:10.1371/journal.pone.0031360)
Supplement: Web Appendix S1 — 224 included systematic reviews. (DOC) [file pone.0031360.s001.doc]

**Web appendix S1: 224 included systematic reviews**

1. Ottawa Panel. (2004) Ottawa Panel Evidence-Based Clinical Practice Guidelines for Electrotherapy and Thermotherapy Interventions in the Management of Rheumatoid Arthritis in Adults. Phys Ther 84: 1016-1043.

2. Adab N, Tudur SC, Vinten J, Williamson P, Winterbottom J (2004) Common antiepileptic drugs in pregnancy in women with epilepsy. Cochrane Database Syst Rev: CD004848.

3. Ahmed N, Ahmedzai S, Vora V, Hillam S, Paz S (2004) Supportive care for patients with gastrointestinal cancer. Cochrane Database Syst Rev: CD003445.

4. Ahovuo-Saloranta A, Hiiri A, Nordblad A, Worthington H, Makela M (2004) Pit and fissure sealants for preventing dental decay in the permanent teeth of children and adolescents. Cochrane Database Syst Rev: CD001830.

5. Albert X, Huertas I, Pereiro, II, Sanfelix J, Gosalbes V, et al. (2004) Antibiotics for preventing recurrent urinary tract infection in non-pregnant women. Cochrane Database Syst Rev: CD001209.

6. Allen D, Dunn L (2004) Aciclovir or valaciclovir for Bell's palsy (idiopathic facial paralysis). Cochrane Database Syst Rev: CD001869.

7. Aronson N, Lefevre F, Piper M, Mark D, Bohn R, et al. (2001) Management of chronic asthma. Evid Rep Technol Assess (Summ): 1-10.

8. Arowojolu AO, Gallo MF, Grimes DA, Garner SE (2004) Combined oral contraceptive pills for treatment of acne. Cochrane Database Syst Rev: CD004425.

9. Attin T, Hannig C, Wiegand A, Attin R (2004) Effect of bleaching on restorative materials and restorations--a systematic review. Dent Mater 20: 852-861.

10. Ball P, Stahlmann R, Kubin R, Choudhri S, Owens R (2004) Safety profile of oral and intravenous moxifloxacin: cumulative data from clinical trials and postmarketing studies. Clin Ther 26: 940-950.

11. Barks L (2004) Therapeutic positioning, wheelchair seating, and pulmonary function of children with cerebral palsy: a research synthesis. Rehabil Nurs 29: 146-153.

12. Baroletti S, Bencivenga GA, Gabardi S (2004) Treating gout in kidney transplant recipients. Prog Transplant 14: 143-147.

13. Basan A, Kissling W, Leucht S (2004) Valproate as an adjunct to antipsychotics for schizophrenia: a systematic review of randomized trials. Schizophr Res 70: 33-37.

14. Bath PM (2004) Theophylline, aminophylline, caffeine and analogues for acute ischaemic stroke. Cochrane Database Syst Rev: CD000211.

15. Bath PM (2004) Prostacyclin and analogues for acute ischaemic stroke. Cochrane Database Syst Rev: CD000177.

16. Bath PM, Bath-Hextall FJ (2004) Pentoxifylline, propentofylline and pentifylline for acute ischaemic stroke. Cochrane Database Syst Rev: CD000162.

17. Bebb JR, Scott BB (2004) How effective are the usual treatments for ulcerative colitis? Aliment Pharmacol Ther 20: 143-149.

18. Bebb JR, Scott BB (2004) How effective are the usual treatments for Crohn's disease? Aliment Pharmacol Ther 20: 151-159.

19. Benson PE, Parkin N, Millett DT, Dyer FE, Vine S, et al. (2004) Fluorides for the prevention of white spots on teeth during fixed brace treatment. Cochrane Database Syst Rev: CD003809.

20. Blake D, Proctor M, Johnson N, Olive D (2005) Cleavage stage versus blastocyst stage embryo transfer in assisted conception. Cochrane Database Syst Rev: CD002118.

21. Bohlius J, Langensiepen S, Schwarzer G, Seidenfeld J, Piper M, et al. (2004) Erythropoietin for patients with malignant disease. Cochrane Database Syst Rev: CD003407.

22. Bohlius J, Reiser M, Schwarzer G, Engert A (2004) Granulopoiesis-stimulating factors to prevent adverse effects in the treatment of malignant lymphoma. Cochrane Database Syst Rev: CD003189.

23. Bolton DA, Cauraugh JH, Hausenblas HA (2004) Electromyogram-triggered neuromuscular stimulation and stroke motor recovery of arm/hand functions: a meta-analysis. J Neurol Sci 223: 121-127.

24. Bongioanni P, Reali C, Sogos V (2004) Ciliary neurotrophic factor (CNTF) for amyotrophic lateral sclerosis/motor neuron disease. Cochrane Database Syst Rev: CD004302.

25. Boomsma CM, Heineman MJ, Cohlen BJ, Farquhar C (2004) Semen preparation techniques for intrauterine insemination. Cochrane Database Syst Rev: CD004507.

26. Brady F (2004) Contextual interference: a meta-analytic study. Percept Mot Skills 99: 116-126.

27. Briel M, Studer M, Glass TR, Bucher HC (2004) Effects of statins on stroke prevention in patients with and without coronary heart disease: a meta-analysis of randomized controlled trials. Am J Med 117: 596-606.

28. Bronfort G, Nilsson N, Haas M, Evans R, Goldsmith CH, et al. (2004) Non-invasive physical treatments for chronic/recurrent headache. Cochrane Database Syst Rev: CD001878.

29. Brosseau L, Welch V, Wells G, DeBie R, Gam A, et al. (2004) Low level laser therapy (Classes I, II and III) for treating osteoarthritis. Cochrane Database Syst Rev: CD002046.

30. Brylewski J, Duggan L (2004) Antipsychotic medication for challenging behaviour in people with learning disability. Cochrane Database Syst Rev: CD000377.

31. Bunn F, Roberts I, Tasker R, Akpa E (2004) Hypertonic versus near isotonic crystalloid for fluid resuscitation in critically ill patients. Cochrane Database Syst Rev: CD002045.

32. Cantineau AE, Cohlen BJ, Al-Inany H, Heineman MJ (2004) Intrauterine insemination versus fallopian tube sperm perfusion for non tubal infertility. Cochrane Database Syst Rev: CD001502.

33. Carlberg B, Samuelsson O, Lindholm LH (2004) Atenolol in hypertension: is it a wise choice? Lancet 364: 1684-1689.

34. Carrick S, Ghersi D, Wilcken N, Simes J (2004) Platinum containing regimens for metastatic breast cancer. Cochrane Database Syst Rev: CD003374.

35. Carter-Pokras O, O'Neill MJ, Cheanvechai V, Menis M, Fan T, et al. (2004) Providing linguistically appropriate services to persons with limited English proficiency: a needs and resources investigation. Am J Manag Care 10 Spec No: SP29-36.

36. Chard DJ, Vaughn S, Tyler BJ (2002) A synthesis of research on effective interventions for building reading fluency with elementary students with learning disabilities. J Learn Disabil 35: 386-406.

37. Chen J, Liu C (2004) Methotrexate for ankylosing spondylitis. Cochrane Database Syst Rev: CD004524.

38. Chen W, Gluud C (2004) Glucocorticosteroids for primary sclerosing cholangitis. Cochrane Database Syst Rev: CD004036.

39. Cheng AC, Stephens DP, Currie BJ (2004) Granulocyte-Colony Stimulating Factor (G-CSF) as an adjunct to antibiotics in the treatment of pneumonia in adults. Cochrane Database Syst Rev: CD004400.

40. Cheng L, Gulmezoglu AM, Oel CJ, Piaggio G, Ezcurra E, et al. (2004) Interventions for emergency contraception. Cochrane Database Syst Rev: CD001324.

41. Chronicle E, Mulleners W (2004) Anticonvulsant drugs for migraine prophylaxis. Cochrane Database Syst Rev: CD003226.

42. Conaty S, Watson L, Dinnes J, Waugh N (2004) The effectiveness of pneumococcal polysaccharide vaccines in adults: a systematic review of observational studies and comparison with results from randomised controlled trials. Vaccine 22: 3214-3224.

43. Cook LA, Vliet H, Pun A, Gallo MF (2004) Vasectomy occlusion techniques for male sterilization. Cochrane Database Syst Rev: CD003991.

44. Coon JT, Ernst E (2004) Complementary and alternative therapies in the treatment of chronic hepatitis C: a systematic review. J Hepatol 40: 491-500.

45. Cordeiro NJ, Oniyangi O (2004) Phytomedicines (medicines derived from plants) for sickle cell disease. Cochrane Database Syst Rev: CD004448.

46. Creutzig A, Lehmacher W, Elze M (2004) Meta-analysis of randomised controlled prostaglandin E1 studies in peripheral arterial occlusive disease stages III and IV. VASA 33: 137-144.

47. Cullum N, McInnes E, Bell-Syer SE, Legood R (2004) Support surfaces for pressure ulcer prevention. Cochrane Database Syst Rev: CD001735.

48. Dalziel K, Round A, Stein K, Garside R, Price A (2004) Effectiveness and cost-effectiveness of imatinib for first-line treatment of chronic myeloid leukaemia in chronic phase: a systematic review and economic analysis. Health Technol Assess 8: iii, 1-120.

49. Davis PG, Tan A, O'Donnell CP, Schulze A (2004) Resuscitation of newborn infants with 100% oxygen or air: a systematic review and meta-analysis. Lancet 364: 1329-1333.

50. Dawson MY, Michalak EE, Waraich P, Anderson JE, Lam RW (2004) Is remission of depressive symptoms in primary care a realistic goal? A meta-analysis. BMC Fam Pract 5: 19.

51. Daya S, Gunby J (2004) Luteal phase support in assisted reproduction cycles. Cochrane Database Syst Rev: CD004830.

52. de Silva A, Jones PW, Spencer SA (2004) Does human milk reduce infection rates in preterm infants? A systematic review. Arch Dis Child Fetal Neonatal Ed 89: F509-513.

53. Decadt B, Siriwardena AK (2004) Radiofrequency ablation of liver tumours: systematic review. Lancet Oncol 5: 550-560.

54. Demicheli V, Rivetti D, Deeks JJ, Jefferson TO (2004) Vaccines for preventing influenza in healthy adults. Cochrane Database Syst Rev: CD001269.

55. Dimmick S, Badawi N, Randell T (2004) Thyroid hormone supplementation for the prevention of morbidity and mortality in infants undergoing cardiac surgery. Cochrane Database Syst Rev: CD004220.

56. Dinh-Zarr T, Goss C, Heitman E, Roberts I, DiGuiseppi C (2004) Interventions for preventing injuries in problem drinkers. Cochrane Database Syst Rev: CD001857.

57. Diwan A, Rardin CR, Kohli N (2004) Uterine preservation during surgery for uterovaginal prolapse: a review. Int Urogynecol J Pelvic Floor Dysfunct 15: 286-292.

58. Ebbert JO, Rowland LC, Montori V, Vickers KS, Erwin PC, et al. (2004) Interventions for smokeless tobacco use cessation. Cochrane Database Syst Rev: CD004306.

59. Edmonds M, McGuire H, Price J (2004) Exercise therapy for chronic fatigue syndrome. Cochrane Database Syst Rev: CD003200.

60. Edwards PS, Lipp A, Holmes A (2004) Preoperative skin antiseptics for preventing surgical wound infections after clean surgery. Cochrane Database Syst Rev: CD003949.

61. Ejere H, Alhassan MB, Rabiu M (2004) Face washing promotion for preventing active trachoma. Cochrane Database Syst Rev: CD003659.

62. Elbaum B, Vaughn S (2003) For which students with learning disabilities are self-concept interventions effective? J Learn Disabil 36: 101-108; discussion 149-150.

63. Elit L, Chambers A, Fyles A, Covens A, Carey M, et al. (2004) Systematic review of adjuvant care for women with Stage I ovarian carcinoma. Cancer 101: 1926-1935.

64. Ernst E, Schmidt K (2004) Homotoxicology--a review of randomised clinical trials. Eur J Clin Pharmacol 60: 299-306.

65. Esposito M, Worthington HV, Thomsen P, Coulthard P (2004) Interventions for replacing missing teeth: different times for loading dental implants. Cochrane Database Syst Rev: CD003878.

66. Esposito M, Worthington HV, Thomsen P, Coulthard P (2004) Interventions for replacing missing teeth: maintaining health around dental implants. Cochrane Database Syst Rev: CD003069.

67. Evers JL, Collins JA (2004) Surgery or embolisation for varicocele in subfertile men. Cochrane Database Syst Rev: CD000479.

68. Fiocchi A, Bouygue GR, Martelli A, Terracciano L, Sarratud T (2004) Dietary treatment of childhood atopic eczema/dermatitis syndrome (AEDS). Allergy 59 Suppl 78: 78-85.

69. Fisher P, van Haselen R, Hardy K, Berkovitz S, McCarney R (2004) Effectiveness gaps: a new concept for evaluating health service and research needs applied to complementary and alternative medicine. J Altern Complement Med 10: 627-632.

70. Flynn CA, Griffin GH, Schultz JK (2004) Decongestants and antihistamines for acute otitis media in children. Cochrane Database Syst Rev: CD001727.

71. Fokkinga WA, Kreulen CM, Vallittu PK, Creugers NH (2004) A structured analysis of in vitro failure loads and failure modes of fiber, metal, and ceramic post-and-core systems. Int J Prosthodont 17: 476-482.

72. Fransen M, Neal B (2004) Non-steroidal anti-inflammatory drugs for preventing heterotopic bone formation after hip arthroplasty. Cochrane Database Syst Rev: CD001160.

73. Freedman KB, Smith AP, Romeo AA, Cole BJ, Bach BR, Jr. (2004) Open Bankart repair versus arthroscopic repair with transglenoid sutures or bioabsorbable tacks for Recurrent Anterior instability of the shoulder: a meta-analysis. Am J Sports Med 32: 1520-1527.

74. French R, Van Vliet H, Cowan F, Mansour D, Morris S, et al. (2004) Hormonally impregnated intrauterine systems (IUSs) versus other forms of reversible contraceptives as effective methods of preventing pregnancy. Cochrane Database Syst Rev: CD001776.

75. Fretheim A, Oxman AD, Flottorp S (2004) Improving prescribing of antihypertensive and cholesterol-lowering drugs: a method for identifying and addressing barriers to change. BMC Health Serv Res 4: 23.

76. Fung MF, Bryson P, Johnston M, Chambers A (2004) Screening postmenopausal women for ovarian cancer: a systematic review. J Obstet Gynaecol Can 26: 717-728.

77. Gaunekar NN, Crowther CA (2004) Maintenance therapy with calcium channel blockers for preventing preterm birth after threatened preterm labour. Cochrane Database Syst Rev: CD004071.

78. Gibson RC, Fenton M, Coutinho ES, Campbell C (2004) Zuclopenthixol acetate for acute schizophrenia and similar serious mental illnesses. Cochrane Database Syst Rev: CD000525.

79. Glenny AM, Hooper L, Shaw WC, Reilly S, Kasem S, et al. (2004) Feeding interventions for growth and development in infants with cleft lip, cleft palate or cleft lip and palate. Cochrane Database Syst Rev: CD003315.

80. Gold C, Voracek M, Wigram T (2004) Effects of music therapy for children and adolescents with psychopathology: a meta-analysis. J Child Psychol Psychiatry 45: 1054-1063.

81. Goldenberg DL, Burckhardt C, Crofford L (2004) Management of fibromyalgia syndrome. JAMA 292: 2388-2395.

82. Gotzsche PC, Johansen HK (2004) Short-term low-dose corticosteroids vs placebo and nonsteroidal antiinflammatory drugs in rheumatoid arthritis. Cochrane Database Syst Rev: CD000189.

83. Gourlay SG, Stead LF, Benowitz NL (2004) Clonidine for smoking cessation. Cochrane Database Syst Rev: CD000058.

84. Green JM, Hewison J, Bekker HL, Bryant LD, Cuckle HS (2004) Psychosocial aspects of genetic screening of pregnant women and newborns: a systematic review. Health Technol Assess 8: iii, ix-x, 1-109.

85. Grimes D, Gallo M, Grigorieva V, Nanda K, Schulz K (2004) Steroid hormones for contraception in men. Cochrane Database Syst Rev: CD004316.

86. Grossman P, Niemann L, Schmidt S, Walach H (2004) Mindfulness-based stress reduction and health benefits. A meta-analysis. J Psychosom Res 57: 35-43.

87. Gubitz G, Sandercock P, Counsell C (2004) Anticoagulants for acute ischaemic stroke. Cochrane Database Syst Rev: CD000024.

88. Gundry S, Wright J, Conroy R (2004) A systematic review of the health outcomes related to household water quality in developing countries. J Water Health 2: 1-13.

89. Hackett ML, Anderson CS, House AO (2004) Interventions for treating depression after stroke. Cochrane Database Syst Rev: CD003437.

90. Hajek P, Stead LF (2004) Aversive smoking for smoking cessation. Cochrane Database Syst Rev: CD000546.

91. Hall NJ, Van Der Zee J, Tan HL, Pierro A (2004) Meta-analysis of laparoscopic versus open pyloromyotomy. Ann Surg 240: 774-778.

92. Han A, Robinson V, Judd M, Taixiang W, Wells G, et al. (2004) Tai chi for treating rheumatoid arthritis. Cochrane Database Syst Rev: CD004849.

93. Handoll HH, Vaghela MV (2004) Interventions for treating mallet finger injuries. Cochrane Database Syst Rev: CD004574.

94. Hardy SC, Riding G, Abidia A (2004) Surgery for deep venous incompetence. Cochrane Database Syst Rev: CD001097.

95. Hawes J, McEwan P, McGuire W (2004) Nasal versus oral route for placing feeding tubes in preterm or low birth weight infants. Cochrane Database Syst Rev: CD003952.

96. Hay PJ, Bacaltchuk J, Stefano S (2004) Psychotherapy for bulimia nervosa and binging. Cochrane Database Syst Rev: CD000562.

97. He FJ, MacGregor GA (2004) Effect of longer-term modest salt reduction on blood pressure. Cochrane Database Syst Rev: CD004937.

98. Heintjes E, Berger MY, Bierma-Zeinstra SM, Bernsen RM, Verhaar JA, et al. (2004) Pharmacotherapy for patellofemoral pain syndrome. Cochrane Database Syst Rev: CD003470.

99. Heisel O, Heisel R, Balshaw R, Keown P (2004) New onset diabetes mellitus in patients receiving calcineurin inhibitors: a systematic review and meta-analysis. Am J Transplant 4: 583-595.

100. Henna MR, Del Nero RG, Sampaio CZ, Atallah AN, Schettini ST, et al. (2004) Hormonal cryptorchidism therapy: systematic review with metanalysis of randomized clinical trials. Pediatr Surg Int 20: 357-359.

101. Henry C, Ghaemi SN (2004) Insight in psychosis: a systematic review of treatment interventions. Psychopathology 37: 194-199.

102. Hesse M (2004) Achieving abstinence by treating depression in the presence of substance-use disorders. Addict Behav 29: 1137-1141.

103. Heyn P, Abreu BC, Ottenbacher KJ (2004) The effects of exercise training on elderly persons with cognitive impairment and dementia: a meta-analysis. Arch Phys Med Rehabil 85: 1694-1704.

104. Hoenig H, Siebens H (2004) Research agenda for geriatric rehabilitation. Am J Phys Med Rehabil 83: 858-866.

105. Holmes KK, Levine R, Weaver M (2004) Effectiveness of condoms in preventing sexually transmitted infections. Bull World Health Organ 82: 454-461.

106. Hooper L, Brown TJ, Elliott R, Payne K, Roberts C, et al. (2004) The effectiveness of five strategies for the prevention of gastrointestinal toxicity induced by non-steroidal anti-inflammatory drugs: systematic review. BMJ 329: 948.

107. Hrobjartsson A, Gotzsche PC (2004) Placebo interventions for all clinical conditions. Cochrane Database Syst Rev: CD003974.

108. Inglis GD, Davies MW (2004) Prophylactic antibiotics to reduce morbidity and mortality in neonates with umbilical artery catheters. Cochrane Database Syst Rev: CD004697.

109. Izquierdo de Santiago A, Khan M (2007) Hypnosis for schizophrenia. Cochrane Database Syst Rev: CD004160.

110. Jardine LA, Jenkins-Manning S, Davies MW (2004) Albumin infusion for low serum albumin in preterm newborn infants. Cochrane Database Syst Rev: CD004208.

111. Jefferson T, Deeks JJ, Demicheli V, Rivetti D, Rudin M (2004) Amantadine and rimantadine for preventing and treating influenza A in adults. Cochrane Database Syst Rev: CD001169.

112. Johnson NP, Mak W, Sowter MC (2004) Surgical treatment for tubal disease in women due to undergo in vitro fertilisation. Cochrane Database Syst Rev: CD002125.

113. Jones M, Schenkel B, Just J, Fallowfield L (2004) Epoetin alfa improves quality of life in patients with cancer: results of metaanalysis. Cancer 101: 1720-1732.

114. Jorm AF, Christensen H, Griffiths KM, Parslow RA, Rodgers B, et al. (2004) Effectiveness of complementary and self-help treatments for anxiety disorders. Med J Aust 181: S29-46.

115. Kenworthy T, Adams CE, Bilby C, Brooks-Gordon B, Fenton M (2004) Psychological interventions for those who have sexually offended or are at risk of offending. Cochrane Database Syst Rev: CD004858.

116. Khan RJ, Fick D, Brammar TJ, Crawford J, Parker MJ (2004) Interventions for treating acute Achilles tendon ruptures. Cochrane Database Syst Rev: CD003674.

117. Kim AH, Vaughn S, Wanzek J, Wei S (2004) Graphic organizers and their effects on the reading comprehension of students with LD: a synthesis of research. J Learn Disabil 37: 105-118.

118. Kinnett D (2004) Botulinum toxin A injections in children: technique and dosing issues. Am J Phys Med Rehabil 83: S59-64.

119. Knudsen UB, Tabor A, Mosgaard B, Andersen ES, Kjer JJ, et al. (2004) Management of ovarian cysts. Acta Obstet Gynecol Scand 83: 1012-1021.

120. Krebs EE, Ensrud KE, MacDonald R, Wilt TJ (2004) Phytoestrogens for treatment of menopausal symptoms: a systematic review. Obstet Gynecol 104: 824-836.

121. Kukuruzovic RH, Elliott EE, O'Loughlin EV, Markowitz JE (2004) Non-surgical interventions for eosinophilic oesophagitis. Cochrane Database Syst Rev: CD004065.

122. Kulier R, Boulvain M, Walker D, Candolle G, Campana A (2004) Minilaparotomy and endoscopic techniques for tubal sterilisation. Cochrane Database Syst Rev: CD001328.

123. Lee A, Done ML (2004) Stimulation of the wrist acupuncture point P6 for preventing postoperative nausea and vomiting. Cochrane Database Syst Rev: CD003281.

124. Lees J, Manning N, Rawlings B (2004) A culture of enquiry: research evidence and the therapeutic community. Psychiatr Q 75: 279-294.

125. Leontiadis GI, McIntyre L, Sharma VK, Howden CW (2004) Proton pump inhibitor treatment for acute peptic ulcer bleeding. Cochrane Database Syst Rev: CD002094.

126. Lesi A, Meremikwu M (2004) High first dose quinine regimen for treating severe malaria. Cochrane Database Syst Rev: CD003341.

127. Lethaby A, Suckling J, Barlow D, Farquhar CM, Jepson RG, et al. (2004) Hormone replacement therapy in postmenopausal women: endometrial hyperplasia and irregular bleeding. Cochrane Database Syst Rev: CD000402.

128. Lewis A, Lookinland S, Beckstrand RL, Tiedeman ME (2004) Treatment of hypertriglyceridemia with omega-3 fatty acids: a systematic review. J Am Acad Nurse Pract 16: 384-395.

129. Liabsuetrakul T, Choobun T, Peeyananjarassri K, Islam M (2004) Antibiotic prophylaxis for operative vaginal delivery. Cochrane Database Syst Rev: CD004455.

130. Lip GY, Felmeden DC (2004) Antiplatelet agents and anticoagulants for hypertension. Cochrane Database Syst Rev: CD003186.

131. Liu JP, Yang M, Du XM (2004) Herbal medicines for viral myocarditis. Cochrane Database Syst Rev: CD003711.

132. Liu JP, Zhang M, Wang WY, Grimsgaard S (2004) Chinese herbal medicines for type 2 diabetes mellitus. Cochrane Database Syst Rev: CD003642.

133. Liu PY, Swerdloff RS, Veldhuis JD (2004) Clinical review 171: The rationale, efficacy and safety of androgen therapy in older men: future research and current practice recommendations. J Clin Endocrinol Metab 89: 4789-4796.

134. Lodi G, Sardella A, Bez C, Demarosi F, Carrassi A (2004) Interventions for treating oral leukoplakia. Cochrane Database Syst Rev: CD001829.

135. Mailis-Gagnon A, Furlan AD, Sandoval JA, Taylor R (2004) Spinal cord stimulation for chronic pain. Cochrane Database Syst Rev: CD003783.

136. Maitra N, Kulier R, Bloemenkamp KW, Helmerhorst FM, Gulmezoglu AM (2004) Progestogens in combined oral contraceptives for contraception. Cochrane Database Syst Rev: CD004861.

137. Manns B, Stevens L, Miskulin D, Owen WF, Jr., Winkelmayer WC, et al. (2004) A systematic review of sevelamer in ESRD and an analysis of its potential economic impact in Canada and the United States. Kidney Int 66: 1239-1247.

138. Matthews SJ, McCoy C (2004) Peginterferon alfa-2a: a review of approved and investigational uses. Clin Ther 26: 991-1025.

139. Mattick RP, Kimber J, Breen C, Davoli M (2004) Buprenorphine maintenance versus placebo or methadone maintenance for opioid dependence. Cochrane Database Syst Rev: CD002207.

140. Matzel KE, Stadelmaier U, Hohenberger W (2004) Innovations in fecal incontinence: sacral nerve stimulation. Dis Colon Rectum 47: 1720-1728.

141. McCarney RW, Lasserson TJ, Linde K, Brinkhaus B (2004) An overview of two Cochrane systematic reviews of complementary treatments for chronic asthma: acupuncture and homeopathy. Respir Med 98: 687-696.

142. McCord JF, Michelinakis G (2004) Systematic review of the evidence supporting intra-oral maxillofacial prosthodontic care. Eur J Prosthodont Restor Dent 12: 129-135.

143. Mehndiratta MM, Hughes RA, Agarwal P (2004) Plasma exchange for chronic inflammatory demyelinating polyradiculoneuropathy. Cochrane Database Syst Rev: CD003906.

144. Montori VM, Helgemoe PK, Guyatt GH, Dean DS, Leung TW, et al. (2004) Telecare for patients with type 1 diabetes and inadequate glycemic control: a randomized controlled trial and meta-analysis. Diabetes Care 27: 1088-1094.

145. Moore H, Summerbell C, Hooper L, Cruickshank K, Vyas A, et al. (2004) Dietary advice for treatment of type 2 diabetes mellitus in adults. Cochrane Database Syst Rev: CD004097.

146. Mowatt G, Vale L, Brazzelli M, Hernandez R, Murray A, et al. (2004) Systematic review of the effectiveness and cost-effectiveness, and economic evaluation, of myocardial perfusion scintigraphy for the diagnosis and management of angina and myocardial infarction. Health Technol Assess 8: iii-iv, 1-207.

147. Mullner M, Urbanek B, Havel C, Losert H, Waechter F, et al. (2004) Vasopressors for shock. Cochrane Database Syst Rev: CD003709.

148. Munkarah AR, Coleman RL (2004) Critical evaluation of secondary cytoreduction in recurrent ovarian cancer. Gynecol Oncol 95: 273-280.

149. Namaka M, Gramlich CR, Ruhlen D, Melanson M, Sutton I, et al. (2004) A treatment algorithm for neuropathic pain. Clin Ther 26: 951-979.

150. Nannini L, Cates CJ, Lasserson TJ, Poole P (2004) Combined corticosteroid and long acting beta-agonist in one inhaler for chronic obstructive pulmonary disease. Cochrane Database Syst Rev: CD003794.

151. Nicopoullos JD, Gilling-Smith C, Almeida PA, Norman-Taylor J, Grace I, et al. (2004) Use of surgical sperm retrieval in azoospermic men: a meta-analysis. Fertil Steril 82: 691-701.

152. Nowak A, Findlay M, Culjak G, Stockler M (2004) Tamoxifen for hepatocellular carcinoma. Cochrane Database Syst Rev: CD001024.

153. Nygaard IE, McCreery R, Brubaker L, Connolly A, Cundiff G, et al. (2004) Abdominal sacrocolpopexy: a comprehensive review. Obstet Gynecol 104: 805-823.

154. Opperman AM, Venter CS, Oosthuizen W, Thompson RL, Vorster HH (2004) Meta-analysis of the health effects of using the glycaemic index in meal-planning. Br J Nutr 92: 367-381.

155. Osborn DA, Sinn J (2004) Soy formula for prevention of allergy and food intolerance in infants. Cochrane Database Syst Rev: CD003741.

156. O'Shea SD, Taylor NF, Paratz J (2004) Peripheral muscle strength training in COPD: a systematic review. Chest 126: 903-914.

157. Pachler J, Wille-Jorgensen P (2004) Quality of life after rectal resection for cancer, with or without permanent colostomy. Cochrane Database Syst Rev: CD004323.

158. Padwal R, Li SK, Lau DC (2004) Long-term pharmacotherapy for obesity and overweight. Cochrane Database Syst Rev: CD004094.

159. Pakos EE, Ioannidis JP (2004) Radiotherapy vs. nonsteroidal anti-inflammatory drugs for the prevention of heterotopic ossification after major hip procedures: a meta-analysis of randomized trials. Int J Radiat Oncol Biol Phys 60: 888-895.

160. Park EW, Schultz JK, Tudiver F, Campbell T, Becker L (2004) Enhancing partner support to improve smoking cessation. Cochrane Database Syst Rev: CD002928.

161. Parker MJ, Gillespie LD, Gillespie WJ (2004) Hip protectors for preventing hip fractures in the elderly. Cochrane Database Syst Rev: CD001255.

162. Patel H, Platt R, Lozano JM, Wang EE (2004) Glucocorticoids for acute viral bronchiolitis in infants and young children. Cochrane Database Syst Rev: CD004878.

163. Pittas AG, Siegel RD, Lau J (2004) Insulin therapy for critically ill hospitalized patients: a meta-analysis of randomized controlled trials. Arch Intern Med 164: 2005-2011.

164. Playford EG, Webster AC, Sorell TC, Craig JC (2004) Antifungal agents for preventing fungal infections in solid organ transplant recipients. Cochrane Database Syst Rev: CD004291.

165. Proctor ML, Hing W, Johnson TC, Murphy PA (2004) Spinal manipulation for primary and secondary dysmenorrhoea. Cochrane Database Syst Rev: CD002119.

166. Qiang W, Ke Z (2004) Water for preventing urinary calculi. Cochrane Database Syst Rev: CD004292.

167. Quigley C (2004) Opioid switching to improve pain relief and drug tolerability. Cochrane Database Syst Rev: CD004847.

168. Quinlivan R, Beynon RJ (2004) Pharmacological and nutritional treatment for McArdle's disease (Glycogen Storage Disease type V). Cochrane Database Syst Rev: CD003458.

169. Ram FS, Ardern KD (2004) Dietary salt reduction or exclusion for allergic asthma. Cochrane Database Syst Rev: CD000436.

170. Ram FS, Picot J, Lightowler J, Wedzicha JA (2004) Non-invasive positive pressure ventilation for treatment of respiratory failure due to exacerbations of chronic obstructive pulmonary disease. Cochrane Database Syst Rev: CD004104.

171. Ram FS, Rowe BH, Kaur B (2004) Vitamin C supplementation for asthma. Cochrane Database Syst Rev: CD000993.

172. Ramakrishnan U, Aburto N, McCabe G, Martorell R (2004) Multimicronutrient interventions but not vitamin a or iron interventions alone improve child growth: results of 3 meta-analyses. J Nutr 134: 2592-2602.

173. Ramirez PT, Frumovitz M, Bodurka DC, Sun CC, Levenback C (2004) Hormonal therapy for the management of grade 1 endometrial adenocarcinoma: a literature review. Gynecol Oncol 95: 133-138.

174. Rees K, Taylor RS, Singh S, Coats AJ, Ebrahim S (2004) Exercise based rehabilitation for heart failure. Cochrane Database Syst Rev: CD003331.

175. Rickard GD, Richardson R, Johnson T, McColl D, Hooper L (2004) Ozone therapy for the treatment of dental caries. Cochrane Database Syst Rev: CD004153.

176. Roberts TG, Jr., Goulart BH, Squitieri L, Stallings SC, Halpern EF, et al. (2004) Trends in the risks and benefits to patients with cancer participating in phase 1 clinical trials. JAMA 292: 2130-2140.

177. Rossetti S, Actis GC, Fadda M, Rizzetto M, Palmo A (2004) The use of the anti-tumour necrosis factor monoclonal antibody--infliximab--to treat ulcerative colitis: implications and trends beyond the available data. Dig Liver Dis 36: 426-431.

178. Roy P, Kumar D (2004) Strictureplasty. Br J Surg 91: 1428-1437.

179. Royle P, Waugh N, McAuley L, McIntyre L, Thomas S (2004) Inhaled insulin in diabetes mellitus. Cochrane Database Syst Rev: CD003890.

180. Rubenstein JH, Laine L (2004) Systematic review: the hepatotoxicity of non-steroidal anti-inflammatory drugs. Aliment Pharmacol Ther 20: 373-380.

181. Saab S, Nieto JM, Ly D, Runyon BA (2004) TIPS versus paracentesis for cirrhotic patients with refractory ascites. Cochrane Database Syst Rev: CD004889.

182. Safer DJ (2004) A comparison of risperidone-induced weight gain across the age span. J Clin Psychopharmacol 24: 429-436.

183. Saunders Y, Ross JR, Broadley KE, Edmonds PM, Patel S (2004) Systematic review of bisphosphonates for hypercalcaemia of malignancy. Palliat Med 18: 418-431.

184. Schroth RJ, Hitchon CA, Uhanova J, Noreddin A, Taback SP, et al. (2004) Hepatitis B vaccination for patients with chronic renal failure. Cochrane Database Syst Rev: CD003775.

185. Scott I, Chan J, Aroney C, Carroll G (2004) Local thrombolysis or rapid transfer for primary angioplasty for patients presenting with ST segment elevation myocardial infarction to hospitals without angioplasty facilities. Intern Med J 34: 373-377.

186. Sedrakyan A, van der Meulen J, Lewsey J, Treasure T (2004) Video assisted thoracic surgery for treatment of pneumothorax and lung resections: systematic review of randomised clinical trials. BMJ 329: 1008.

187. Seferiadis A, Rosenfeld M, Gunnarsson R (2004) A review of treatment interventions in whiplash-associated disorders. Eur Spine J 13: 387-397.

188. Shakiba H, Dinesh S, Anne MK (2004) Advanced trauma life support training for hospital staff. Cochrane Database Syst Rev: CD004173.

189. Shekelle PG, Morton SC, Maglione M, Suttorp M, Tu W, et al. (2004) Pharmacological and surgical treatment of obesity. Evid Rep Technol Assess (Summ): 1-6.

190. Silagy C, Lancaster T, Stead L, Mant D, Fowler G (2004) Nicotine replacement therapy for smoking cessation. Cochrane Database Syst Rev: CD000146.

191. Sommerfield T, Hiatt WR (2004) Omega-3 fatty acids for intermittent claudication. Cochrane Database Syst Rev: CD003833.

192. Soo S, Moayyedi P, Deeks J, Delaney B, Lewis M, et al. (2004) Psychological interventions for non-ulcer dyspepsia. Cochrane Database Syst Rev: CD002301.

193. Spinks AB, Wasiak J, Villanueva EV, Bernath V (2004) Scopolamine for preventing and treating motion sickness. Cochrane Database Syst Rev: CD002851.

194. Stade B, Shah V, Ohlsson A (2004) Vaginal chlorhexidine during labour to prevent early-onset neonatal group B streptococcal infection. Cochrane Database Syst Rev: CD003520.

195. Steed H, Chapman W, Laframboise S (2004) Endometriosis-associated ovarian cancer: a clinicopathologic review. J Obstet Gynaecol Can 26: 709-715.

196. Stevens B, Yamada J, Ohlsson A (2004) Sucrose for analgesia in newborn infants undergoing painful procedures. Cochrane Database Syst Rev: CD001069.

197. Stevens TP, Blennow M, Soll RF (2004) Early surfactant administration with brief ventilation vs selective surfactant and continued mechanical ventilation for preterm infants with or at risk for respiratory distress syndrome. Cochrane Database Syst Rev: CD003063.

198. Swanson HL (1999) Reading research for students with LD: a meta-analysis of intervention outcomes. J Learn Disabil 32: 504-532.

199. Swanson HL, Sachse-Lee C (2000) A meta-analysis of single-subject-design intervention research for students with LD. J Learn Disabil 33: 114-136.

200. Tan A, Schulze A, O'Donnell CP, Davis PG (2004) Air versus oxygen for resuscitation of infants at birth. Cochrane Database Syst Rev: CD002273.

201. Taverner D, Latte J, Draper M (2004) Nasal decongestants for the common cold. Cochrane Database Syst Rev: CD001953.

202. Thomson CE, Gibson JN, Martin D (2004) Interventions for the treatment of Morton's neuroma. Cochrane Database Syst Rev: CD003118.

203. Trinh KV, Phillips SD, Ho E, Damsma K (2004) Acupuncture for the alleviation of lateral epicondyle pain: a systematic review. Rheumatology (Oxford) 43: 1085-1090.

204. Turner C, Spinks A, McClure R, Nixon J (2004) Community-based interventions for the prevention of burns and scalds in children. Cochrane Database Syst Rev: CD004335.

205. Twetman S, Petersson L, Axelsson S, Dahlgren H, Holm AK, et al. (2004) Caries-preventive effect of sodium fluoride mouthrinses: a systematic review of controlled clinical trials. Acta Odontol Scand 62: 223-230.

206. Uitterhoeve RJ, Vernooy M, Litjens M, Potting K, Bensing J, et al. (2004) Psychosocial interventions for patients with advanced cancer - a systematic review of the literature. Br J Cancer 91: 1050-1062.

207. van der Lugt JC, Rozing PM (2004) Systematic review of primary total elbow prostheses used for the rheumatoid elbow. Clin Rheumatol 23: 291-298.

208. Verschuur HP, de Wever WW, van Benthem PP (2004) Antibiotic prophylaxis in clean and clean-contaminated ear surgery. Cochrane Database Syst Rev: CD003996.

209. Villanueva E, Bennett MH, Wasiak J, Lehm JP (2004) Hyperbaric oxygen therapy for thermal burns. Cochrane Database Syst Rev: CD004727.

210. Vincent JL, Navickis RJ, Wilkes MM (2004) Morbidity in hospitalized patients receiving human albumin: a meta-analysis of randomized, controlled trials. Crit Care Med 32: 2029-2038.

211. Vink AC, Birks JS, Bruinsma MS, Scholten RJ (2004) Music therapy for people with dementia. Cochrane Database Syst Rev: CD003477.

212. Walker S, Monteil M, Phelan K, Lasserson TJ, Walters EH (2004) Anti-IgE for chronic asthma in adults and children. Cochrane Database Syst Rev: CD003559.

213. Wark PA, Gibson PG, Wilson AJ (2004) Azoles for allergic bronchopulmonary aspergillosis associated with asthma. Cochrane Database Syst Rev: CD001108.

214. Wasiak J, Hoare B, Wallen M (2004) Botulinum toxin A as an adjunct to treatment in the management of the upper limb in children with spastic cerebral palsy. Cochrane Database Syst Rev: CD003469.

215. Watts CC, Whurr R, Nye C (2004) Botulinum toxin injections for the treatment of spasmodic dysphonia. Cochrane Database Syst Rev: CD004327.

216. Wendel-Vos GC, Schuit AJ, Feskens EJ, Boshuizen HC, Verschuren WM, et al. (2004) Physical activity and stroke. A meta-analysis of observational data. Int J Epidemiol 33: 787-798.

217. Westby M, Benson M, Gibson P (2004) Anticholinergic agents for chronic asthma in adults. Cochrane Database Syst Rev: CD003269.

218. Wheeler DM, Vimalachandra D, Hodson EM, Roy LP, Smith GH, et al. (2004) Interventions for primary vesicoureteric reflux. Cochrane Database Syst Rev: CD001532.

219. Wong D, Adams CE, David A, Quraishi SN (2004) Depot bromperidol decanoate for schizophrenia. Cochrane Database Syst Rev: CD001719.

220. Woodgate P, Flenady V, Steer P (2004) Intramuscular penicillin for the prevention of early onset group B streptococcal infection in newborn infants. Cochrane Database Syst Rev: CD003667.

221. Yap C, Furness S, Farquhar C (2004) Pre and post operative medical therapy for endometriosis surgery. Cochrane Database Syst Rev: CD003678.

222. Yoshizumi WM, Tsourounis C (2004) Effects of creatine supplementation on renal function. J Herb Pharmacother 4: 1-7.

223. Zhang FK (2004) Interferon-alfa in the treatment of chronic hepatitis B. Hepatobiliary Pancreat Dis Int 3: 337-340.

224. Zupan J, Garner P, Omari AA (2004) Topical umbilical cord care at birth. Cochrane Database Syst Rev: CD001057.
