# Supplementary material for: Does Consideration and Assessment of Effects on Health Equity Affect the Conclusions of Systematic Reviews? A Methodology Study
Source: PLoS One. 2012 Mar 13;7(3):e31360. doi: 10.1371/journal.pone.0031360 (PMC3302723; doi:10.1371/journal.pone.0031360)
Supplement: Web Appendix S2 — Systematic reviews (n = 29) which assessed differences across PROGRESS-Plus by: 1) subgroup analysis; 2) targeted; or 3) description of individual studies. (DOCX) [file pone.0031360.s002.docx]

Web Appendix S2: Systematic reviews (n=29) which assessed differences across PROGRESS-Plus by: 1) subgroup analysis; 2) targeted; or 3) description of individual studies

| **Reference** | **Intervention** | **Population** | **Method** | **Health inequalities hypothesized to affect intervention effects** | **PROGRESS-Plus Factor** | **Finding** | **Implications related to vulnerable populations** |
| --- | --- | --- | --- | --- | --- | --- | --- |
| **[1]** | Maxillofacial prosthodontic care for oral cancer | People with oral cancer | Subgroup analysis- Descriptive | No | Gender, SES | Gender, SES had no significant impact on outcomes; | Research needed to assess statistical power of effect modifiers (e.g. age, marital status, smoking status) |
| **[2]** | Genetic screening for pregnant women | Pregnant women | Subgroup analysis- Descriptive | No | SES | Videos may be more effective than leaflets for those who cannot read. | Research is needed on interventions for low SES to reduce inequalities in understanding and anxiety |
| **[3]** | Permanent colostomy after rectal resection for cancer | People with rectal resection for cancer | Subgroup analysis- Descriptive | Yes | Education, Gender, Occupation, Religion and SES | One study included mainly patients with low social class, low income level and poor education and it is argued that patients in poor areas may have problems managing their stoma | Research needed: Inadequate reporting of social class in studies needs to be addressed |
| **[4]** | Tamoxifen for hepatocellular carcinoma | People with hepatocellular carcinoma | Subgroup analysis- Descriptive | No | Gender | Describe influence of gender in 2 trials: 1 trial no difference, 1 trial found benefit for men but not women | Not done |
| **[5]]** | Clonidine for smoking cessation | Smokers | Subgroup analysis- Descriptive | No | Gender | 4 studies showed greater effect of clonidine for women | Not done |
| **[6]** | Moxifloxacin for respiratory tract infections in adults | Respiratory infection | Subgroup analysis- Descriptive | No | Gender, Plus-age | In individual studies, predisposition to adverse effects was associated with greater age and female sex | Effective for women and men, and for elderly |
| **[7]** | Non-steroidal anti-inflammatory drugs | People taking NSAIDs | Subgroup analysis- Descriptive | No | Gender, Plus-Age | Older patients at higher risk for liver injury; 2 studies found no effect of gender, 1 study found men at increased risk | Effective for women and men |
| **[8]** | Myocardial perfusion scintography for diagnosis and management of angina and myocardial infarction | People with angina or myocardial infarction | Subgroup analysis- Descriptive | Yes | Gender | 6 studies which assessed gender concluded that SPECT provides important independent prediction of survival in both men and women | Effective for women and men (no difference) |
| **[9]** | Interventions for children with learning disabilities | Students (children or adults) with learning disabilities | Subgroup analysis-pooled results | No | Gender | No difference between males and females | Effective for both males and females (no difference in effects) |
| **[10]** | Fluoride mouthrinses | Non-selected populations of all ages | Subgroup analysis-pooled results | Yes | SES (background fluoride exposure as a proxy for SES) | 0.95 per year difference in DMF; 23% preventive fraction difference | More effective for low SES due to higher baseline risk of caries |
| **[11]** | Reading interventions | Children with learning disabilities | Subgroup analysis-pooled results | No | Gender | Effect size was 0.48 greater for studies which did not report the gender ratio compared to those that did | Implications not discussed |
| **[12]** | Exercise training to prevent stroke | General | Subgroup analysis-pooled results | No | Gender | Relative risk of stroke 0.54 for men, 0.76 for women, difference = 0.22 (p=0.07) | No implications related to gender |
| **[13]** | Micronutrient interventions | Children | Subgroup analysis-pooled results | No | SES (measured by proxy of baseline nutritional status- height and weight for age) | Multi-micronutrient supplementation has positive effect on child growth | Need research on food-based approaches that are sustainable and feasible in resource poor settings |
| **[14]** | Peg interferon Alfa-2a | Chronic hepatitis C virus infection | Subgroup analysis -pooled results | Yes | Race/ethnicity | Blacks have lower sustained viral response than white (15% vs 35% in one study and 26% vs 39% in another study) | More research is needed on blacks |
| **[15]** | Pneumococcal polysaccharide vaccines to prevent pneumonia | Adults | Subgroup analysis-pooled results | No | Adults in LMIC vs. elderly in HIC | RCTs in LMIC where prevalence is high show benefit (RR 0.66, 95% CI: 0.57-0.77). RCTs in elderly show no effect (RR 1.03, 95% CI: 0.86-1.25) | Properly conducted randomized trials are needed to support the use of conjugate vaccines in the elderly |
| **[16]** | Hip protectors | Elderly | Targeted | No | Plus- frail elderly | Evidence of effectiveness of hip protectors in institutional settings with high background incidence of hip fracture | Effective for frail elderly |
| **[17]** | Exercise training to improve function and cognition in elderly with cognitive impairment | Elderly people (>65 yrs) with cognitive impairment | Targeted | Yes | Plus- elderly with cognitive impairment | Exercise training improves fitness, physical function and cognitive function in elderly with dementia | exercise beneficial for elderly with cognitive impairment |
| **[18]** | Psychological interventions for those at risk of offending | People who have sexually offended or are at risk of offending, , population described as vulnerable | Targeted | No | Plus- sexual offenders | Psychological interventions can be studies in RCTs. Limited evidence of their benefits for this population | ethics of providing this unproven treatment to vulnerable people outside of a trial is debatable |
| **[19]** | Face-washing promotion for preventing active trachoma | Trachoma endemic communities (i.e. Africa, Asia, Middle East) | Targeted | No | Plus- LMIC endemic countries | Face-washing combined with antibiotics reduces severe trachoma | Not done |
| **[20]** | Buprenorphine maintenance vs. placebo or methadone maintenance for opioid dependence | Opioid dependence | Targeted | No | Plus- Heroine-addicted drug users | Buprenoprphine is effective, but not as effective as methadone | Different intervention may be preferred for some vulnerable populations due to feasibility : Buprenorphine may have advantage when alternate day dosing is desirable due to feasibility issues |
| **[21]** | Interventions for preventing injuries in problem drinkers | Problem drinkers | Targeted | No | Plus- problem drinkers | Interventions reduce injuries and their antecedents | Not done |
| **[22]** | Antipsychotic medication for challenging behaviour in people with learning disability | People with learning disability | Targeted | Yes | Plus- disease status of learning disability described as "culturally abnormal behaviours" | No evidence on whether these medications help or harm adults with learning disability and challenging behaviours | Research needed: Policy: managers should insist on good quality research, clinicians should use judgment and clinical experience |
| **[23]** | Mindfulness-based stress reduction for chronic illness | Chronically ill | Targeted | No | Plus- disability of chronic illness | Stress reduction beneficial on mental and physical health outcomes with effect size of 0.5 | Not done |
| **[24]** | Providing linguistically appropriate services | People with limited English proficiency | Targeted | Yes | Race/ethnicity- language, disadvantaged non-English speakers | Linguistic interpretation results in higher adherence and better health status | Different interventions for non-English speakers: procedures needed to identify patients who need linguistic interpreters |
| **[25]** | Household water treatment and storage interventions to reduce diarrhea | People in LMIC | Targeted | Yes | Plus- LMIC | Household treatment reduces cholera but not diarrhea | Need to assess factors that affect post-source water quality (e.g. including hygiene education, household water treatment methods) |
| **[26]** | Therapeutic communities | Mainly disadvantaged prison inmates or drug abusers | Targeted | No | Plus- drug abusers or prison inmates | Confirmed effectiveness of therapeutic communities on success (e.g. reduction in criminal behavior) | Not done |
| **[27]** | Treatment interventions to improve insight in psychosis | Psychosis | Targeted | No | Plus- Disease status of schizophrenia | Possible benefits of psycho-education | Not done |
| **[28]** | Psychosocial treatment + antidepressants to promote abstinence from drugs and alcohol | Alcohol or drug dependent people | Targeted | No | Plus- alcohol or drug dependency | No support for claim that psychosocial interventions can enhance effectiveness of antidepressants | Not done |
| **[29]** | Complementary and alternative therapies for hepatitis c | Hepatitis c virus infection | Targeted | No | Plus- disease status of HCV results in discrimination due to association with injection drug users and homeless | No evidence for the use of complementary therapies in subgroups who have been denied interferon-alpha therapy in the past (e.g. HIV co-infection or psychiatric conditions) | Not done |

**Web Appendix S2 References**

1. McCord JF, Michelinakis G (2004) Systematic review of the evidence supporting intra-oral maxillofacial prosthodontic care. European Journal of Prosthodontics & Restorative Dentistry 12: 129-135.

2. Green JM, Hewison J, Bekker HL, Bryant LD, Cuckle HS (2001) Psychosocial aspects of genetic screening of pregnant women and newborns: a systematic review. Health Technology Assessment (Winchester, England) 8: iii-iix.

3. Pachler J, Wille-Jorgensen P (2005) Quality of life after rectal resection for cancer, with or without permanent colostomy. Cochrane Database of Systematic Reviews Art. No. CD004323.

4. Nowak A, Findlay M, Culjak G, Stockler M (2004) Tamoxifen for hepatocellular carcinoma. Cochrane Database of Systematic Reviews CD001024.

5. Gourlay SG, Stead LF, Benowitz NL (2004) Clonidine for smoking cessation. Cochrane Database of Systematic Reviews CD000058.

6. Ball P, Stahlman R, Kubin R, Choudhri S, Owens R (2004) Safety profile of oral and intravenous moxifloxacin: cumulative data from clinical trials and postmarketing studies. Clin Ther 26: 940-950.

7. Rubenstein JH, Laine L (2004) Systematic review: the hepatotoxicity of non-steroidal anti-inflammatory drugs. Aliment Pharmacol Ther 20: 373-380.

8. Mowatt G, Vale L, Brazzelli M, Hernandez R, Murray A, et al. (2001) Systematic review of the effectiveness and cost-effectiveness, and economic evaluation, of myocardial perfusion scintigraphy for the diagnosis and management of angina and myocardial infarction. [Review] [138 refs]. Health Technology Assessment (Winchester, England) 8: iii-iiv.

9. Swanson HL, Sachse-Lee C (2000) A meta-analysis of single-subject-design intervention research for students with LD. Journal of learning disabilities 33: 114-136.

10. Twetman S, Petersson L, Axelsson S, Dahlgren H, Holm AK, et al. (2004) Caries-preventive effect of sodium fluoride mouthrinses: a systematic review of controlled clinical trials. [Review] [79 refs]. Acta Odontologica Scandinavica 62: 223-230.

11. Swanson HL (1999) Reading research for students with LD: a meta-analysis of intervention outcomes. Journal of learning disabilities 32: 504-532.

12. Wendel-Vos GC, Schuit AJ, Feskens EJ, Boshuizen HC, Verschuren WM, Saris WH, Kromhout D (2004) Physical activity and stroke. A meta-analysis of observational data. Int J Epidemiol 33: 787-798.

13. Ramakrishnan U, Aburto N, McCabe G, Martorell R (2004) Multimicronutrient interventions but not vitamin a or iron interventions alone improve child growth: results of 3 meta-analyses. [Review] [80 refs]. Journal of Nutrition 134: 2592-2602.

14. Matthews SJ, McCoy C (2004) Peginterferon alfa-2a: a review of approved and investigational uses. [Review] [149 refs]. Clinical Therapeutics 26: 991-1025.

15. Conaty S, Watson L, Dinnes J, Waugh N (2004) The effectiveness of pneumococcal polysaccharide vaccines in adults: a systematic review of observational studies and comparison with results from randomised controlled trials. Vaccine 22: 3214-3224.

16. Parker MJ, Gillespie LD, Gillespie WJ (2005) Hip protectors for preventing hip fractures in the elderly. Cochrane Database of Systematic Reviews CD001255.

17. Heyn P, Abreu BC, Ottenbacher KJ (2004) The effects of exercise training on elderly persons with cognitive impairment and dementia: a meta-analysis. Archives of Physical Medicine & Rehabilitation 85: 1694-1704.

18. Kenworthy T, Adams CE, Bilby C, Brooks-Gordon B, Fenton M (2008) Psychological interventions for those who have sexually offended or are at risk of offending. Cochrane Database of Systematic Reviews CD004858.

19. Ejere H, Alhassan MB, Rabiu M (2004) Face washing promotion for preventing active trachoma. Cochrane Database of Systematic Reviews CD003659.

20. Mattick RP, Kimber J, Breen C, Davoli M (2003) Buprenorphine maintenance versus placebo or methadone maintenance for opioid dependence. Cochrane Database of Systematic Reviews Issue 2. Art. No.: CD002207.pub2. DOI: 10.1002/14651858.CD002207.

21. Dinh-Zarr T, Goss C, Heitman E, Roberts I, DiGuiseppi C (2004) Interventions for preventing injuries in problem drinkers. Cochrane Database of Systematic Reviews CD001857.

22. Brylewski J, Duggan L (2004) Antipsychotic medication for challenging behaviour in people with learning disability. Cochrane Database of Systematic Reviews CD000377.

23. Grossman P, Niemann L, Schmidt S, Walach H (2004) Mindfulness-based stress reduction and health benefits. A meta-analysis. Journal of Psychosomatic Research 57: 35-43.

24. Carter-Pokras O, O'Neill MJ, Cheanvechai V, Menis M, Fan T, et al. (2004) Providing linguistically appropriate services to persons with limited English proficiency: a needs and resources investigation. American Journal of Managed Care 10: Spec-36.

25. Gundry S, Wright J, Conroy R (2004) A systematic review of the health outcomes related to household water quality in developing countries. Journal of Water and Health 2: 1-13.

26. Lees J, Manning N, Rawlings B (2004) A culture of enquiry: research evidence and the therapeutic community. Psychiatric Quarterly 75: 279-294.

27. Henry C, Ghaemi SN (2004) Insight in psychosis: a systematic review of treatment interventions. Psychopathology 37: 194-199.

28. Hesse M (2004) Achieving abstinence by treating depression in the presence of substance-use disorders. Addictive Behaviors 29: 1137-1141.

29. Coon JT, Ernst E (2004) Complementary and alternative therapies in the treatment of chronic hepatitis C: a systematic review. Journal of Hepatology 40: 491-500.
